# Identification of Structural Features of Hydrocinnamic Acid Related to Its Allelopathic Activity against the Parasitic Weed *Cuscuta campestris*

**DOI:** 10.3390/plants11212846

**Published:** 2022-10-26

**Authors:** Antonio Moreno-Robles, Antonio Cala Peralta, Jesús G. Zorrilla, Gabriele Soriano, Marco Masi, Susana Vilariño-Rodríguez, Alessio Cimmino, Mónica Fernández-Aparicio

**Affiliations:** 1 Campus de Rabanales, University of Córdoba, 14071 Córdoba, Spain; 2 Department of Chemical Sciences, University of Naples Federico II, Complesso Universitario Monte S. Angelo, Via Cintia, 80126 Naples, Italy; 3 Allelopathy Group, Department of Organic Chemistry, Facultad de Ciencias, Institute of Biomolecules (INBIO), University of Cadiz, C/Avenida República Saharaui, s/n, 11510 Puerto Real, Spain; 4 ALGOSUR S.A., Ctra Lebrija-Trebujena km 5.5, 41740 Sevilla, Spain; 5 Department of Plant Breeding, Institute for Sustainable Agriculture (IAS), CSIC, Avenida Menéndez Pidal s/n, 14004 Córdoba, Spain

**Keywords:** field dodder, parasitic weeds, phenylpropanoic acid, allelochemicals, structure–activity relationship, sustainable crop protection

## Abstract

*Cuscuta campestris* is a parasitic weed species that inflicts worldwide noxious effects in many broadleaf crops due to its capacity to withdraw nutrients and water directly from the crop vascular system using haustorial connections. *Cuscuta campestris* control in the majority of crops affected is non-existent, and thus, research for the development of control methods is needed. Hydrocinnamic acid occurs naturally in the rhizosphere, playing regulatory roles in plant–plant and plant–microbe communities. The toxicity of hydrocinnamic acid against *C. campestris* was recently identified. In the present work, a structure–activity relationship study of 21 hydrocinnamic acid analogues was performed to identify key structural features needed for its allelopathic action against the seedling growth of this parasitic plant. The findings of this study provide the first step for the design of herbicides with enhanced activity for the control of *C. campestris* infection.

## 1. Introduction

More than 1% of flowering plants have evolved the capacity to parasitize other plants to obtain all or part of their required nutrients [1]. Among them, the dodders (*Cuscuta* genus) contain over 170 species distributed across tropical, subtropical, and temperate regions [1,2]. *Cuscuta campestris* is considered one of the most damaging parasitic weed species, severely affecting the yield of dicotyledonous crops of economic importance [3]. There is no effective *Cuscuta* control for the most affected crops [4,5]. After *Cuscuta* germination, a filiform seedling twines around the nearest crop stem producing haustoria, which penetrate into the crop stem and fuse with the crop vascular system to extract nutrients and water [6,7]. *Cuscuta* plants are root- and leaf-less plants with little or no chlorophyll, and therefore, they are completely dependent on crop-derived nutrition, otherwise they die within 7 to 10 days after germination [8]. The identification of allelochemicals that target the vulnerable pre-attached *Cuscuta* seedlings and interfere with the necessary host contact for infection is the first step for the design of alternatives that provide efficacy and sustainability to parasitic weed chemical control [7]. 

During a recent screening to discover new inhibitors of *Cuscuta* seedling growth, hydrocinnamic acid was identified as a promising compound showing strong inhibitory activity [9]. Hydrocinnamic acid, also known as phenylpropanoic acid, is a carboxylic acid with the formula C_9_H_10_O_2_ belonging to the class of phenylpropanoids that originate from the shikimic acid pathway [10]. Hydrocinnamic acid occurs naturally in the rhizosphere, either originating during the breakdown of crop residues [11] or exuded in intact form from plant roots [12], regulating ecosystems through different roles in plant–plant [13] and plant–microbe interactions [14]. Hydrocinnamic acid has been reported to inhibit seed germination in several species [15,16,17]. Thus, the objective of this work was to study the structure–activity relationships of 21 hydrocinnamic acid analogues in order to identify key structural features needed for its herbicidal action against *Cuscuta*. This is the first report on the phytotoxic activity of many of the tested compounds.

## 2. Results and Discussion

**Figure 1:**
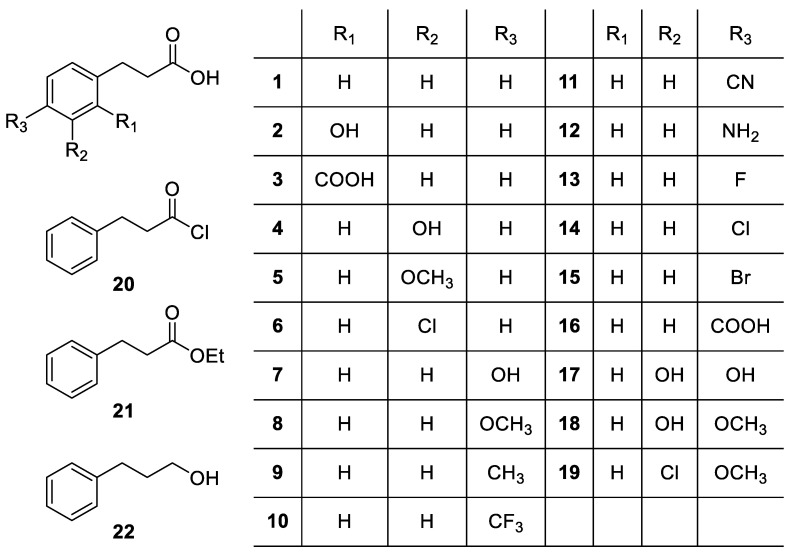
Summary of the structures of compounds **1**–**22** used in this study.

The *Cuscuta* growth inhibition of hydrocinnamic acid (**1**, Figure 1) was studied in vitro in comparison with 21 structural analogues (**2**–**22**, Figure 1) in a range of concentrations from 0.25 to 1 mM. 

**Figure 2:**
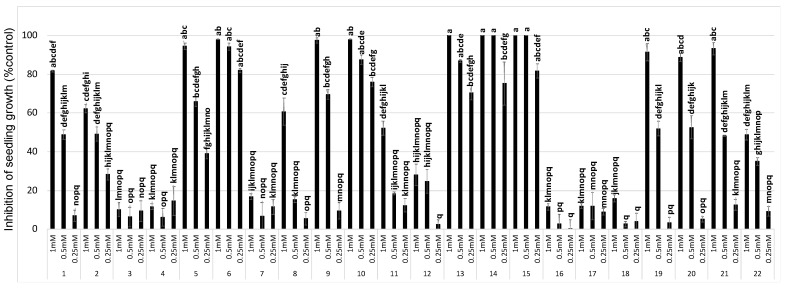
In vitro assessment of the *Cuscuta* growth inhibition induced by compounds **1**–**22** at concentrations of 1, 0.5, and 0.25 mM. Bars with different letters are significantly different (Tukey test at *p* < 0.05). Error bars represent the standard error of the mean.

Five days after treatment, *Cuscuta* growth was significantly affected by the compound treatment (ANOVA, *p* < 0.001) by the concentration applied (ANOVA, *p* < 0.001), and by the interaction of compound x concentration (ANOVA, *p* < 0.001). Figure 2 shows different levels of activity among hydrocinnamic acid (**1**) and its analogues (**2**–**22**), which allowed the classification of compounds with (i) enhanced activity, (ii) similar activity, and (iii) decreased activity in comparison with the parent compound (**1**).

### 2.1. Study of Structure–Activity Relationship on the Growth Inhibition Induced by Hydrocinnamic Acid

The growth inhibition results shown in Figure 2 were used to calculate the IC_50_ values in order to compare the effect of the substitution on the bioactivity, and CLog*p* values were calculated to correlate the activity level with the lipophilicity. These parameters are shown in Table 1.

The strongest bioactivity was found for compounds **6**, **10**, and **13**–**15**, with IC_50_ values less than 250 µM. All of them achieved 100% (or close) inhibition at the highest concentration (1 mM), with compounds **6**, **14**, and **15** maintaining this level of inhibition at 0.5 mM. The key success of these compounds (**6**, **10**, and **13**–**15**) was also their ability to cause inhibition values higher than 75% at the lowest concentration tested (0.25 mM). In this regard, it should be noted that there is a structural correlation between compound **14** and 2,4-dichlorophenoxyacetic acid, one of the most frequently used herbicides due to its efficacy, selectivity, and broad spectrum of weed control [18].

On the other hand, the lowest bioactivity was found for compounds **3**, **4**, **7**, **12**, and **16**–**18**. The bioactivity of these compounds was statistically significant, but below the 50% baseline for the highest concentration tested. This result for compound **7** is in agreement with previous studies that showed moderate or weak phytotoxicity for this compound [19,20], though it should be noted that higher phytotoxicity was previously found on the root growth of tomato and radish [21].

Regarding the correlation between the activity level and the lipophilicity, it was observed that compounds with CLog*p* values lower than 1.712 (Table 1) were found to be the least active. On the other hand, compounds with CLog*p* values higher than 2.046 were found to be the most active. However, no clear direct correlation was found between the linear evolution of CLog*p* and the bioactivity. The relationship of high CLog*p* with bioactivity could be due to the fact that the compounds are preferentially distributed in hydrophobic environments, such as the lipid bilayer of the membrane. This is related to studies where it has been reported that cinnamic acid and derivatives produce changes in the permeability of the cell membrane [22] and reduce H^+^ ATPase activity [23].

In general, by comparison with hydrocinnamic acid (**1**), whose IC_50_ value was 518 μM, the bioactivity was influenced by the position and the type of functional groups it contained. It was observed that depending on the position at the aromatic ring, the effect of the functional group would improve or decrease the bioactivity of hydrocinnamic acid (**1**). Therefore, halogens at para or meta positions would significantly increase the bioactivity (**13**–**15**, and **6**), a methoxy group at meta (**5**) increases the activity of compound **1** while at para (**8**) it would decrease it. In addition, the hydroxy group at ortho (**2**) maintained similar level of activity than compound **1**, while at meta (**4**) and para (**7**), they drastically decreased the bioactivity, even when a second functionalization was included in the form of an extra hydroxy (**17**) or methoxy group (**18**). This low activity observed for compound **17** supports the moderate or low inhibitory activity reported in other studies on the growth of *Triticum aestivum*, *Lactuca sativa*, and *Setaria viridis* [19,24,25].

The detailed analysis of the effects of each functional group on the bioactivity is reported below.

Halogenated substituents increased the inhibitory activity of the core molecule, hydrocinnamic acid, regardless of the position. The compound possessing the largest and least electronegative Br group (**15**) had the strongest inhibitory activity. There appeared to be no significant difference between a substituent Cl at para and meta (**14** and **6**, respectively). Among the halogenated derivatives, the lowest bioactivity was shown by that containing the smallest halogen atom (F, compound **13**). Inclusion of a halogen atom even increased the bioactivity of the weakly active compound **8** (containing a methoxy group), almost doubling it in the case of compound **19**. To contextualize these results regarding the halogenation effects on the bioactivity, it can be highlighted that the phytotoxic activity of several halogenated derivatives of natural compounds has been known for some time. Halogenated derivatives of benzoic acid (weakly phytotoxic) with improved phytotoxicity were reported [26,27], as well as the finding of chlorinated and fluorinated derivatives of benzoxazinones with potent phytotoxicity [28,29]. These last studies described how the position of the halogen atom plays a key role in the level of phytotoxicity as a consequence of the marked electronic transformation of the tested compounds. A study with different chlorinated derivatives of benzophenones also showed that the position of the Cl atom in the aromatic ring is relevant for growth-inhibiting or chlorosis-inducing activities, suggesting the impact of unknown factors, involving steric effects [30]. Given that studies such as those already mentioned have found different levels of activity regarding the position of the Cl atom, it is worth highlighting the results herein reported for hydrocinnamic acid, in which compounds **6** and **14**, differing in the ortho or para positions of the Cl atom, showed similar inhibition on *Cuscuta* growth.

Hydroxyl groups had a negative effect on the bioactivity. There were differences in activity depending on where the alcohol was substituted, being found to induce an improved activity for the ortho derivative (**2**, IC_50_ = 572 μM) greater than the meta (**4**) or para (**7**) derivatives (IC_50_ > 1000 μM). The activity for the para derivative is similar to the activity of the related compound, *p*-coumaric acid, found in a previous study on *Cuscuta* [9]. The increased activity of the hydroxylated derivative at the ortho position (**2**) was also found in a previous study on *Cuscuta* [9], which may be due to its ability to cyclize and form coumarins [31]. Indeed, compound **2** also generates growth inhibition on radish seedlings [21]. In a previous study, scopoletin and umbelliferone were found to have low but significant activity on *Cuscuta* [9]. The cyclization in scopoletin and umbelliferone occurs from cinnamic acid, but an ortho alcohol is necessary for cyclization, and could be also relevant for the increased activity found in hydroxyl at the ortho position.

Methoxy groups had a positive effect on the bioactivity at the meta (**5**) but negative at the para position (**8**). Nevertheless, in both cases, methylation of the corresponding hydroxylated compounds at these positions increased the bioactivity (**4** and **7**). This improvement regarding the hydrocinnamic acids was also previously described for the family of flavones, for which it has been reported that the most active are generally methoxy-substituted [32].

Carboxy groups on the aromatic ring both at para (**16**) and ortho (**3**) positions reduced the activity to negligible levels. Methyl and trifluoromethyl groups at the para position both increased the bioactivity, especially in the case of the CF_3_ group (**10**), which could be related to the similarity in size to hydrogen but the different electronegativity of F. Cyano and amino groups at the para position, on the other hand, substantially decreased the bioactivity of compound **1**, which was more apparent in the case of compound **12** with an amino group.

Carboxylic acid derivatives. Three molecules derived from the carboxylic acid of hydrocinnamic acid were studied: acyl chloride (**20**), ethyl ester (**21**), and alcohol (**22**). Derivatization of hydrocinnamic acid (**1**) to form the ethyl ester (**21**) had little effect on the bioactivity, with very similar EC_50_ (518 and 511 μM, respectively), while the CLog*p* was very different (1.903 and 2.808, respectively). Thus, neither the lipophilicity or the acidity of the compounds appeared to be affecting the bioactivity. This finding might be a key factor, since the control of the solubility and acidity of the bioactive compound by esterification may ease the future formulation of the bioactive compounds. Stronger activity was found for the acyl chloride derivative of **1** (**20**), demonstrating again the beneficial effect of halogens on the bioactivity. In the case of compound **22**, where the acid group has been reduced to an alcohol, the bioactivity was almost lost, demonstrating the importance of the acid group for the growth inhibition activity.

**Figure 3:**
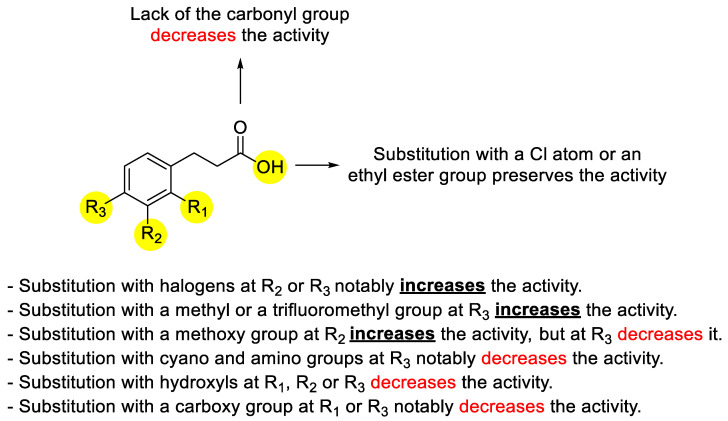
Structure–activity relationship of hydrocinnamic acid regarding the inhibitory activity on *Cuscuta* growth.

The overall structure–activity relationship discussion described above is summarized in Figure 3.

### 2.2. Identification of Inductors of Necrosis in C. campestris Seedlings

**Figure 4:**
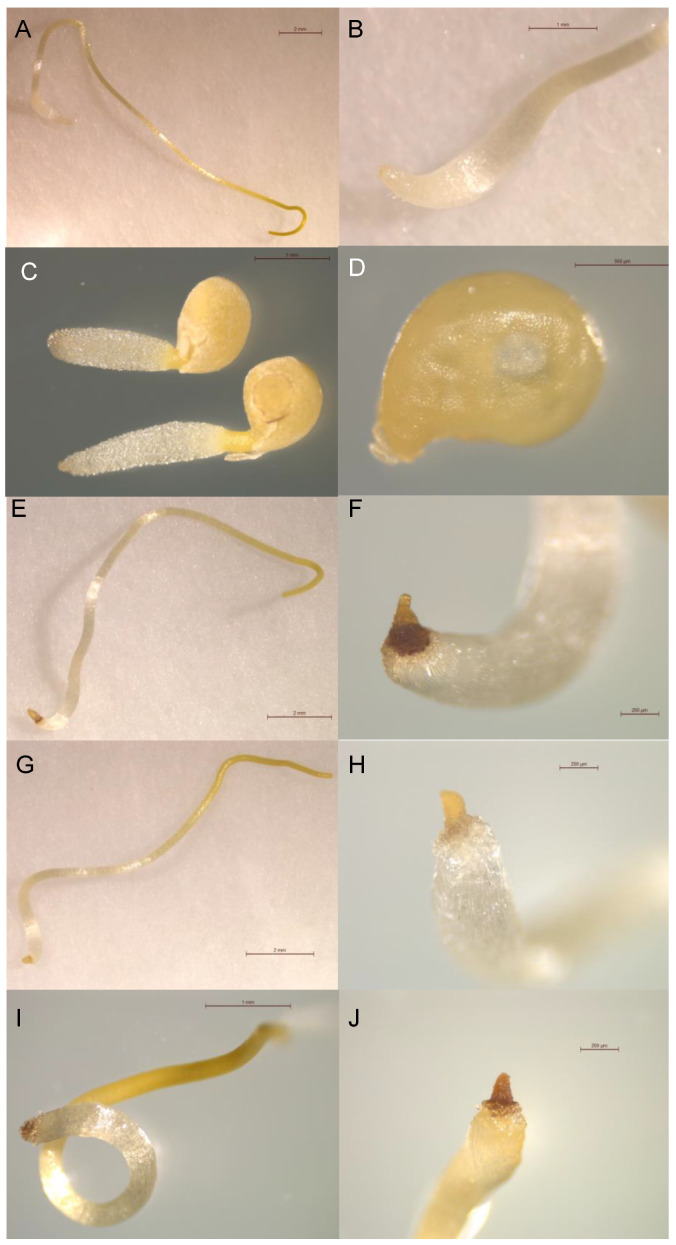
Photographs showing a *Cuscuta* seedling treated with control (**A**,**B**); hydrocinnamic acid (**1**) 1 mM (**C**); 3-(4-fluorophenyl)propionic acid (**13**) (**D**); 3,4-dihydroxyhydrocinnamic acid (**17**) (**E**,**F**); 3-(3-hydroxy-4-methoxyphenyl)propionic acid (**18**) (**G**,**H**); 3-(4-aminophenyl)propionic acid (**12**) (**I**,**J**).

Additional to the modification of inhibitory activity of seedling growth, this SAR study was also used to identify structural features that induced necrosis in *Cuscuta* seedlings (Figure 4). The induction of necrosis observed in this study was significantly affected by the compound treatment (ANOVA, *p* < 0.001). No necrosis was observed in *Cuscuta* seedlings treated with the control or hydrocinnamic acid (Figure 4A–C). Despite not having significant growth-reducing activity, intense necrosis was observed in the root apices of all seedlings treated with compounds **17** and **12** (Figure 4E,F,I,J). Treatments with compound **18** (Figure 4G,H) also induced necrosis, but necrosis was observed to be less intense than the necrosis induced by compounds **12** and **17**. Compound **17** is similar to caffeic acid, the only difference being the absence of the double bond between carbons 2 and 3. It has been observed that, as in compound **17**, caffeic acid does not induce *Cuscuta* growth inhibition, but rather the induction of necrosis [9]. Both compounds with the highest necrosis effect (**12** and **17**) also possess low and similar CLog*p* values (0.676 and 0.639, respectively), lower than in the case of compound **18** (1.085), indicating that low lipophilicity may play a role in producing the necrotic effect. It is interesting to consider that other studies also reported necrotic activity for compound **7** [33,34].

In our previous study, we reported the cinnamic-acid-derived compounds caffeic acid, ferulic acid, vanillic acid, and naringenin, all having necrosis-inducing effects on *Cuscuta* seedlings that varied from strong to moderate [9]. 

By comparing previous findings with the results reported in the present research, some hints about the structural requirements for necrosis-inducing activity can be found. First, the presence of the double bond of cinnamic acid is not mandatory to obtain a strong necrosis-inducing effect, since compound **17** has a similar necrotic effect to caffeic acid. In addition, the presence of two adjacent hydroxy groups in the aromatic ring is beneficial for inducing necrosis, and the methylation of one of these groups decreases the necrotic effect, as observed previously between caffeic and ferulic acid [9], and confirmed in the present work with compound **17** when compared with compound **18**. 

Moreno-Robles et al. [9] reported that vanillic acid, also containing a methoxy group, shows the negative effect of the methylation on the necrosis, while also hints at the relevance of the phenol fragment for necrosis. On the other hand, compound **12**, with an amino group, has a similar effect to the hydroxylated compounds, hinting that polar groups, such as amino and hydroxyl groups, with the possibility to form hydrogen bonds, could have a positive effect on the necrosis-inducing capacity. 

## 3. Materials and Methods

### 3.1. Plant Material and Chemicals

Seeds of *Cuscuta* were collected in July 2019 from mature *Cuscuta campestris* plants parasitizing field pea at the Institute for Sustainable Agriculture (IAS-CSIC), Alameda del Obispo Research Center (Córdoba, southern Spain, coordinates 37.856 N, 4.806 W, datum WGS84). Dry *Cuscuta* seeds were separated from capsules by sifting with a 0.6 mm mesh sieve followed by winnowing with a fan. *Cuscuta* seeds were stored dry in the dark at room temperature until use for this work in 2022.

Hydrocinnamic acid and its 21 analogues were purchased from Sigma-Aldrich (St. Louis, MO, USA): hydrocinnamic acid (**1**, cat. no. 135232), 3-(2-hydroxyphenyl)propionic acid (**2**, cat. no. 393533), 3-(2-carboxyphenyl)propionic acid (**3**, cat. no. 406465), 3-(3-hydroxyphenyl)propanoic acid (**4**, cat. no. PH011597), 3-(3-methoxyphenyl)propionic acid (**5**, cat. no. 349763), 3-(3-chlorophenyl)propionic acid (**6**, cat. no. 631302), 3-(4-hydroxyphenyl)propionic acid (**7**, cat. no. H52406), 3-(4-methoxyphenyl)propanoic acid (**8**, cat. no. M23527), 3-(*p*-tolyl)propionic acid (**9**, cat. no. 118265), 4-(trifluoromethyl)hydrocinnamic acid (**10**, cat. no. 457035), 3-(4-cyanophenyl)propionic acid (**11**, cat. no. 746010), 3-(4-aminophenyl)propionic acid (**12**, cat. no. 560251), 3-(4-fluorophenyl)propionic acid (**13**, cat. no. 560502), 3-(4-chlorophenyl)propionic acid (**14**, cat. no. 656151), 3-(4-bromophenyl)propionic acid (**15**, cat. no. 595438), 3-(4-carboxyphenyl)propionic acid (**16**, cat. no. 531553), 3,4-dihydroxyhydrocinnamic acid (**17**, cat. no. 102601), 3-(3-hydroxy-4-methoxyphenyl)propionic acid (**18**, cat. no. CDS006461), 3-(3-chloro-4-methoxyphenyl)propionic acid (**19**, cat. no. 638773), hydrocinnamoyl chloride (**20**, cat. no. 249440), ethyl 3-phenylpropionate (**21**, cat. no. 284416), 3-phenyl-1-propanol (**22**, cat. no. 140856).

### 3.2. In Vitro Experiments for Screening of Allelopathy against Growth of Cuscuta Seedling

A screening of the 21 compounds (**2**–**22**) described in Figure 1 was performed to identify allelopathic activity against the growth of *Cuscuta* seedling. Seeds of *C. campestris* show physical dormancy induced by a thick seed coat that preserves seedbank viability in agricultural fields over time [7]. To promote *Cuscuta* germination, the hard coat of *Cuscuta* seeds was eliminated by scarification with sulfuric acid for 45 min [35], followed by thorough rinses. Then, five scarified *Cuscuta* seeds were placed using tweezers onto 5 cm diameter filter paper discs inside 5.5 cm diameter Petri dishes. All compounds were dissolved in methanol and then diluted to 1, 0.5, and 0.25 mM in sterilized distilled water. This was conducted for all compounds except for the compound 3-(4-carboxyphenyl)propionic acid, which was dissolved in dimethyl sulfoxide, or the compounds 3-phenyl-1-propanol, ethyl 3-phenylpropionate, and hydrocinnamoyl chloride, which were purchased in liquid formulation and dissolved directly in water. The final concentration of organic solvent in all treatments was 1%, including for the compounds 3-phenyl-1-propanol, ethyl 3-phenylpropionate, and hydrocinnamoyl chloride. Triplicate aliquots of 1 mL of each treatment were applied to filter paper discs containing the scarified *Cuscuta* seeds. Triplicate aliquots of treatment only containing 1% of solvent and sterile distilled water were used as a control. Treated *Cuscuta* seeds were incubated in the dark at 23 °C for 5 days. The seedling length was measured in each of the five *Cuscuta* seedlings for each of the three replicate filter paper discs per treatment. Seedling growth for each treatment was calculated in relation to the seedling growth of the corresponding control. In addition, notes were taken for each *Cuscuta* seedling regarding whether the root apex had developed necrosis. The percentage of seedlings that developed a necrotic root was calculated in each triplicated disk for each treatment. 

### 3.3. Calculations and Statistical Analysis

Compounds that reached inhibitions of 50% and that were active at more than one concentration were statistically analyzed to determine their IC_50_ using GraphPad Prism v.5.00 software package (GraphPad Software, Inc., San Diego, CA, USA). The bioactivity data were fitted to a sigmoidal dose–response model with variable slope. Calculation of CLog*p* was performed using ChemOffice v20.1 (PerkinElmer, Wal-tham, MA, USA) using the appropriate tool in ChemDraw Professional [36]. All bioassays were performed using a completely randomized design. Percentage data were approximated to normal frequency distribution by means of angular transformation. Then, percentage data were subjected to analysis of variance (ANOVA). The significance of mean differences among treatments was evaluated by Tukey test at *p* < 0.05. Statistical analysis was performed using SPSS software 27 (SPSS Inc., Chicago, IL, USA).

## 4. Conclusions

The results demonstrate that phytotoxicity is influenced by the position and the type of functional groups present on the substituted hydrocinnamic acid analogues. In particular, the carbonyl group of the propanoid chain seems an important factor for activity. Furthermore, the presence of halogens on the aromatic ring increases the activity, while substitutions with cyano and amino groups, as well as hydroxyl or carboxyl groups, decreases the activity. Interesting data were also obtained with the presence of a methoxy group in the meta position, while its presence in the para position had a negative effect on the bioactivity. Structural features that induced necrosis in *Cuscuta* seedlings were also identified, and the results suggest that low lipophilicity may play a role in determining the necrotic effect. These results provide interesting and useful information for the design of herbicides for the control of *C. campestris*, starting from the compounds with increased activity in respect to hydrocinnamic acid. However, future studies are needed to determine the mode of action of the active compounds and their ecotoxicity before realizing formulations for practical application as herbicides.

## Figures and Tables

**Table 1:** IC_50_ and CLog*p* values of compounds **1**–**22**, ~1000, inhibition was close to 50% at the highest tested concentration; >1000, compound was far from 50% inhibition at the highest concentration tested but activity was significant; <250, IC_50_ was lower than the lowest tested concentration.

Compound	CLog*p*	IC_50_ (μM)	R^2^	Compound	CLog*p*	IC_50_ (μM)	R^2^
**1**	1.903	518	0.9961	**12**	0.676	>1000	-
**2**	1.186	572	0.9978	**13**	2.046	<250	-
**3**	0.746	>1000	-	**14**	2.616	<250	-
**4**	1.236	>1000	-	**15**	2.766	<250	-
**5**	1.822	334	0.9950	**16**	1.646	>1000	-
**6**	2.616	<250	-	**17**	0.639	>1000	-
**7**	1.236	>1000	-	**18**	1.085	>1000	-
**8**	1.822	879	0.9990	**19**	2.445	488	0.9987
**9**	2.402	415	1.000	**20**	2.228	484	0.9979
**10**	2.786	<250	-	**21**	2.808	511	0.9981
**11**	1.336	~1000	-	**22**	1.712	~1000	-

## Data Availability

Not applicable.
